# DNA Metabarcoding Approach as a Potential Tool for Supporting Official Food Control Programs: A Case Study

**DOI:** 10.3390/foods13182941

**Published:** 2024-09-17

**Authors:** Anna Mottola, Chiara Intermite, Roberta Piredda, Lucilia Lorusso, Lucia Ranieri, Stefania Carpino, Gaetano Vitale Celano, Angela Di Pinto

**Affiliations:** 1 Department of Veterinary Medicine, University of Bari Aldo Moro, Prov. le Casamassima 62, Km 3, Valenzano, 70010 Bari, Italy; anna.mottola@uniba.it (A.M.); chi.inte@gmail.com (C.I.); robpiredda@gmail.com (R.P.); lucia.ranieri@uniba.it (L.R.); gaetanovitale.celano@uniba.it (G.V.C.); angela.dipinto@uniba.it (A.D.P.); 2 Department of Central Inspectorate for Fraud Repression and Quality Protection of the Agri-Food Products and Foodstuffs, Ministry of Agriculture, Food Sovereignty and Forests (ICQRF-MASAF), Via Quintino Sella 42, 00187 Rome, Italy; s.carpino@masaf.gov.it

**Keywords:** food authenticity, NGS, food official controls, meat product

## Abstract

Food authentication significantly impacts consumer health and the credibility of Food Business Operators (FBOs). As European regulations mandate the verification of food authenticity and supply chain integrity, competent authorities require access to innovative analytical methods to identify and prevent food fraud. This study utilizes the DNA metabarcoding approach on meat preparations, sampled during an official control activity. It assesses animal and plant composition by amplifying DNA fragments of the 12S rRNA and trnL (UAA) genes, respectively. The results not only confirmed the declared species but also revealed undeclared and unexpected taxa in products labelled as containing a single animal species and various unspecified plant species. Notable findings such as the presence of Murinae, *Sus scrofa*, *Ovis aries*, and *Pisum sativum* could raise public health concerns, compromise consumer choices made for ethical or religious reasons, and reflect the hygienic conditions of the processing plant. This study demonstrates that the DNA metabarcoding approach looks to be a promising support tool for official control authorities to ensure food authenticity and safety, and to develop risk profiles along the supply chain.

## 1. Introduction

Food authentication issues have become increasingly significant in recent years due to growing concerns over food fraud and mislabeling. Acts of food fraud can have far-reaching effects on consumers and threaten a brand’s integrity. Incidents of food fraud in the past, such as the so-called ‘horsegate’ scandal of 2013, revealed a major breakdown in the traceability of the food supply chain, highlighting the need to verify authenticity so as to protect both the food industry and consumers [1,2,3].

In response to several cases of food fraud, the European Commission introduced the Official Controls Regulation (EU) No. 2017/625 [4], aimed at preventing food fraud through the designation of European Union reference centres for the authenticity and integrity of the agri-food chain. Moreover, in order to apply European legislation effectively, the relevant authorities require access to harmonized data on reported fraud, as well as expertise to help identify vulnerable points in the supply chain and potential violations [5]. Consequently, verifying food authenticity requires the availability of standardized analytical methodologies capable of detecting potentially fraudulent activities in markets where food products are continuously being introduced. For instance, molecular techniques such as real-time PCR are employed in accredited laboratories for authenticating meat products (ISO/TS 20224) [6] or for detecting GMOs [7], while DNA barcoding is used for species identification in fish fillets (UNI CEN/TS 17303) [8]. Despite the cost-effectiveness and efficiency of these methodologies, such approaches are often inadequate for complex food matrices as they can fail to identify multiple species, typically showing only the dominant species [7].

In recent years, DNA metabarcoding has emerged as a promising molecular approach for species identification in complex matrices containing various ingredients and subjected to different production processes, without prior knowledge of the species present. DNA metabarcoding combines DNA barcoding with Next Generation Sequencing (NGS) platforms, enabling the simultaneous amplification and sequencing of DNA from multiple species within the same sample. Several studies have applied DNA metabarcoding to processed seafood, honey, and meat products, showing its ability to detect unexpected and undeclared animal species [8,9,10,11,12,13,14,15].

Considering the need to both update analytical methodologies in the field of food safety and to propose innovative methods for use in official control activities against food fraud, this study aims to investigate the applicability of DNA metabarcoding for the analysis of meat preparations, collected during an official control activity by the Central Inspectorate for Quality Protection and Fraud Repression of Agri-food Products (ICQRF-MASAF). The study assesses both animal and plant compositions in order to verify label information and the traceability system, as a guarantee of an effective food safety management system.

## 2. Materials and Methods

### 2.1. Sample Acceptance

Two prepacked samples, one of swine sausage and one of minced beef, were delivered to the Food Safety Section of the Department of Veterinary Medicine of Valenzano (University of Bari, Italy) (DiMeV-UNIBA) by ICQRF-MASAF to assess the species composition of the products through the DNA metabarcoding approach. Samples were taken frozen to the food safety laboratory, thawed at +4 °C, and subsequently processed. The labels were checked, focusing on the ingredient list for each product.

### 2.2. DNA Extraction, Purification and Sequencing

In order to increase the probability of detecting the various species, two replicates per sample were included. Each replicate was obtained by taking three aliquots of 50 mg from three different points in the same sample. The genomic DNA from each aliquot was extracted and purified using the DNeasy Blood and Tissue Kit (QIAGEN, Hilden, Germany), following the protocol described in [12]. The DNA extracted from each aliquot was then pooled. Negative extraction control (no added sample) was incorporated to verify the purity of the extraction reagents. The purity and concentration of the DNA were assessed by measuring the A260 nm/A280 nm ratio using a BioPhotometer D30 filter (Eppendorf, Milan, Italy). For animal components, the primer pair 12S-V5: 5′-TTAGATACCCCACTATGC-3′ and 12S-V5: 5′-TAGAACAGGCTCCTCTAG-3′ [16] was used to amplify a fragment of ≈98 bp of the 12S rRNA mitochondrial gene. For plant components, the primer pair g: 5′-GGGCAATCCTGAGCCAA-3′ and h: 5′-CCATTGAGTCTCTGCACCTATC-3′ [17] was used to amplify a fragment of ≈40 bp of the chloroplast trnL (UAA) gene. PCR amplifications and the subsequent Illumina paired-end sequencing (2 × 300 bp) on the Illumina MiSeq platform (Illumina, Cambridge, UK) were carried out by IGA Technology Services S.r.l. (Udine, Italy).

### 2.3. Data Processing, Taxonomic Assignment, and Composition Assessment

The Illumina paired-end raw reads were pre-processed to obtain Amplicon Sequence Variants (ASVs) for animal and plant composition using the DADA2 R package (v. 1.22.0) [18]. In brief, the primers were removed and then forward and reverse-trimmed based on their quality scores. The filtered reads were then used to train the error model using a machine learning approach. Forward and reverse reads were then dereplicated to generate unique sequences and denoised (collapsed) into ASVs applying the trained error model. Finally, the forward and reverse reads were merged and checked for chimera sequences. Representative sequences for each ASV were taxonomically assigned by blasting the representative sequences against GenBank in remote mode using the standalone BLAST+ suite [19]. Assignments with a similarity of <90% were discarded, whereas sequences with a similarity in the range of 100–98% or lower than 98% were assigned at the species and genus levels, respectively, and merged [20]. In the event of ambiguous assignments (shared sequence among species), the Lowest Common Ancestor (LCA) approach [21] was applied, meaning that if one read finds matches to different species with the same percentage similarity, it will be assigned to the lowest common taxonomic rank. Subsequently, the number of reads found in the negative control was used as a removal threshold, with taxonomic assignments having totals below the removal threshold being discarded [22]. If the removal threshold was lower than the number of taxonomically assigned reads, it was subtracted from the total. Plots were generated using the R package ggplot2 [23]. Then, molecular identifications of the animal and plant components were compared with the ingredients declared on the labels.

## 3. Results

### 3.1. Animal Composition

The Illumina sequencing for 12S generated a total of 635,719 raw reads that was reduced to 598,720 after filtering, corresponding to a total of five animal taxa. Overall, 99.83% of the total reads were unambiguously assigned at the species level and 0.16% at the subfamily level (Supplementary Table S1).

**Figure 1:**
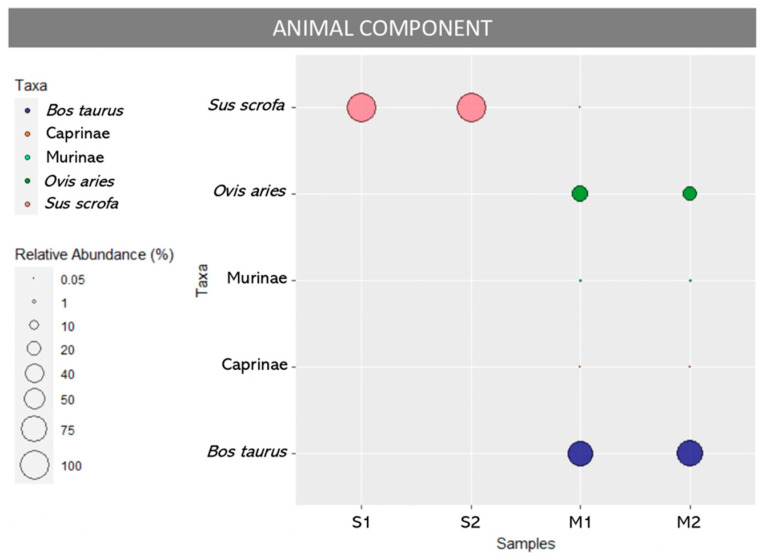
Animal composition in sausage (S1, S2 replicates) and minced meat (M1, M2 replicates) samples. The size of each circle represents the percentage of reads in each replicate over the total reads found in that replicate.

The relative abundance of reads attributed to different taxa is shown in Figure 1 and in Table 1. Both replicates of sample S (S1, S2) contained only *Sus scrofa* (100%), confirming the species declared on the label. In sample M, the replicates (M1, M2) were dominated by *Bos taurus* (69.9% and 79.8%) in combination with *Ovis aries* (29.6% and 19.8%), along with traces of Murinae (0.32% and 0.33%) and Caprinae (0.09% and 0.06%) and, in the M1 replicate alone, also a very small amount of *Sus scrofa* (0.13%).

### 3.2. Plant Composition

The Illumina sequencing for trnL (UAA) generated a total of 391,377 raw reads that was reduced to 202,199 after filtering, corresponding to a total of six plant taxa. Of these, 89.13% of the total reads were assigned at the species level, 10.21% at the genus level, and 0.64% at the subfamily level (Supplementary Table S2).

**Figure 2:**
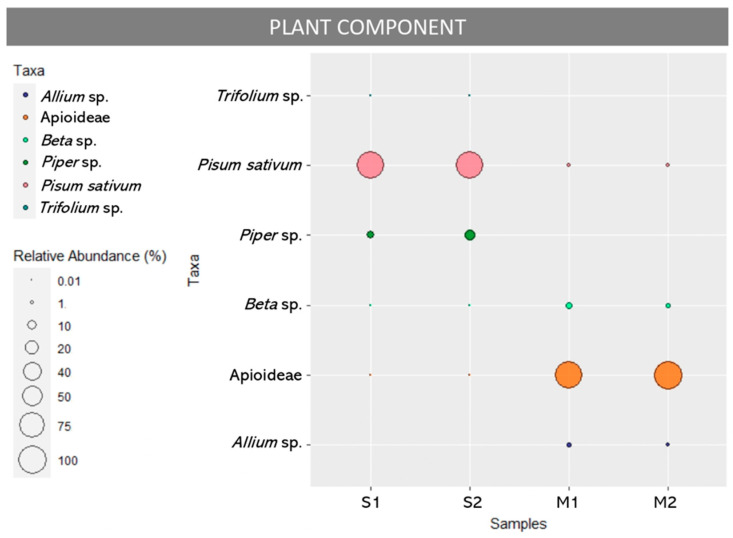
Plant composition in sausage (S1, S2 replicates) and minced meat (M1, M2 replicates) samples. The size of each circle represents the percentage of reads in each replicate over the total reads found in that replicate.

The relative abundance of reads attributed to the different taxa is shown in Figure 2 and in Table 1. Molecular analysis revealed that the main constituent of the sausage samples (S1 and S2) was *Pisum sativum* (93% and 87.2%), followed by *Piper* spp. (6.6% and 12.5%) and traces of *Trifolium* spp. (0.3% and 0.3%), Apioideae (0.04% and 0.04%) and *Beta* spp. (0.01% and 0.01%). The minced beef sample (M1 and M2) was dominated by Apioideae (92% and 95.5%), alongside a small quantity of *Beta* spp. (5% and 2.3%) and *Allium* spp. (2% and 1.3%) and traces of *Pisum sativum* (0.9% and 0.9%).

## 4. Discussion and Conclusions

This study confirms that DNA metabarcoding is of great interest for testing the authenticity of meat-based foods [9,12,13,14]. To the best of our knowledge, this is the first study to apply DNA metabarcoding to simultaneously assess the animal and plant composition of meat products labelled as containing only one animal species and several, unspecified, spices and natural flavourings.

The DNA metabarcoding analysis uncovered the presence of unexpected animal and plant ingredients not declared on the labels. Indeed, the presence of *Ovis aries*, as well as the traces of *Sus scrofa* and Caprinae detected in the study, could be accidental due to poor adherence either to Good Manufacturing Practices (GMP) and/or to equipment cleaning procedures (e.g., knives, workbenches, meat grinders) [24].

Alternatively, the detection of *Ovis aries* could result from the intentional addition of sheep by-products resulting from previous processing [24] or it could have been illegally introduced into production through fraudulent or missing documentation, such as falsified entry or health certificates. Such practices could pose human health risks due to the possible presence of residues of veterinary drugs administered without respecting withdrawal times and correct dosages or from the introduction of diseased animals into the food chain [25,26]. Whatever the scenario, the presence of undeclared animal species can infringe consumer rights and, in particular, may violate consumers’ religious and ethical beliefs that ban the consumption of certain species [27,28,29]. Worryingly, and unexpectedly, both replicates of sample M also showed traces of Murinae (e.g., mouse, rat) (Figure 1). This could indicate poor hygiene and sanitation at the processing plant and, therefore, an inadequate pest control plan, infringing one of the pre-requisites of the Hazard Analysis and Critical Control Point (HACCP) system. Pests such as rodents can contaminate both raw materials and finished products through droppings, urine, and feces, which raises health concerns as they can transmit foodborne pathogens. Indeed, some significant human pathogens, such as *Clostridium perfringens*, *Escherichia coli*, *Listeria monocytogenes*, and *Salmonella* Enteriditis, can be found on their skin and in their digestive systems [30]. Moreover, rats are carriers of leptospirosis, caused by *Leptospira* spp., a major public health concern, especially in developing countries [31]. Consequently, risks such as contact with or the ingestion of water and food contaminated by the bacterium cannot be ruled out, as they can facilitate the spread of this zoonotic disease among meat-handling workers and consumers [32,33].

With regard to the plant component, the presence of *Pisum sativum* detected in this study could be due to attempts either to improve the meat’s texture, stability, and water-binding capacity, or to mask low-quality meat. Indeed, as reported in [34], the use of vegetable proteins is common in meat products, which explains its predominance in sample S. Although pea is not currently listed as an allergen (Annex II of Reg. (EU) 1169/2011 [35]) due to its hypoallergenic properties, three pea allergens have been described and recognized by the International Union of Immunological Sciences. Indeed, a pea allergy featuring symptoms such as asthma, nausea, vomiting, diarrhea, urticaria, and dermatitis has been documented in children and adults. Notably, some children with a known allergy to peanuts have shown severe allergic reactions after the ingestion of pea products. Also, it appears that dried peas, when added in the form of flour and protein isolates, can cause severe allergic reactions compared to green peas due to the higher number of allergenic proteins. Given the above, the addition of peas to the list of allergens should be considered, especially if their use in foods and the prevalence of pea allergies were to increase [36,37]. A further point of discussion concerns the detection of Apioideae, a subfamily of Apiaceae, as the main plant constituent in sample M (Figure 2). This subfamily includes carrot, parsley, coriander, and other plants used as aromatic herbs, spices, and flavourings in meat products and more generally in the food industry. They can be intentionally added for technological purposes to extend the shelf-life of meat products by reducing spoilage. Indeed, they contain phenolic compounds, flavonoids, tannins, and vitamins, which are known for their natural antioxidant properties, and, therefore, are often used in foods to replace artificial ones (e.g., nitrates and nitrites) [38,39]. Furthermore, DNA metabarcoding confirmed the presence of pepper (*Piper* spp.) in the sausage sample, which was explicitly declared on the label as a characteristic ingredient of the product. Also, this approach identified *Allium* spp. in the minced meat sample. Both of these ingredients are commonly used in the food industry due to their antibacterial properties that extend the shelf-life of foods, preventing food spoilage and the proliferation of foodborne pathogens [40]. Specifically, pepper (both white and black) has been observed to retard discoloration in fresh sausages and inhibit lipid oxidation, resulting in a delayed formation of odours [41]. The identification of *Beta* spp. in both samples S and M could suggest the intentional addition of red beetroot, a variety of *Beta vulgaris*, which is used in some meat products as a natural colourant thanks to the presence of betalains, which increase the redness of meats, and hence may help mask quality degradations. In addition, it could be added to meat as it contains bioactive phytochemicals (polyphenols, flavonoids, and other functional antioxidant compounds), thus providing a natural antioxidant solution that can be used to preserve qualities and extend processed meat’s shelf life instead of synthetic nitrates and nitrites [42,43]. Despite the above-mentioned properties, to date its use is not authorized as an additive in meat preparations, such as minced meat and sausages, except for traditional meat products (e.g., ‘merguez-type’, ‘butifarra fresca, ‘kebap’) (Regulation (EU) No. 601/2014 [44]). Finally, the detection of unexpected *Trifolium* spp., a common weed, only in sample S could indicate accidental contamination during harvesting of the spices and herbs [45]. Alternatively, contamination could result from pollen ingress into the production environment.

Unlike the animal component, in this case a comparison between the plant species declared on the label and the molecular identifications was not totally feasible, unless explicitly stated, as in the case of pepper in sample S. Indeed, where spices and herbs do not exceed a total of 2% by weight of the finished product, they may be referred to on the label using the generic term “spices”, so there is no obligation to declare each single plant species (Regulation (EU) No. 1169/2011 [35]). The same applies to flavourings labelled generically as “natural flavourings”, which, according to Regulation (EU) No. 1334/2008 [46], may include so-called “flavour preparations”. The latter can consist of herbs and spices with flavouring properties obtained by traditional methods such as drying and grinding. This shows how difficult it is to draw a demarcation line between flavourings and spices or herbs for some plant species used in the food industry.

From an analytical point of view, several studies have demonstrated the semi-quantitative nature of DNA metabarcoding as the abundance of relative reads can provide an approximation of taxa proportion in complex matrices [13,14,22,47,48]. Different factors such as genome size, copy number for nuclear regions, number of mitochondria in cells/tissues/organs, or primer affinity could affect the number of reads per taxa [49].

Also, although the current Regulation (EU) No. 2017/625 [4] pushes for fraud prevention, some important regulatory gaps are present, such as the lack of threshold limits and details on how to discriminate between accidental or intentional presence. In addition, the study highlights a further gap in the labelling of potential allergens (e.g., *Pisum sativum*) added to meat products, which could potentially increase the likelihood of developing allergies due to increased exposure to them [37].

The acquisition of DNA metabarcoding data would help the adequate implementation of official controls, carried out periodically, based on a risk assessment and with adequate frequency. Indeed, DNA metabarcoding data would make it possible to develop risk profiles and vulnerability for each Food Business Operator (FBO), supply chain and food product by constituting the basis for drawing up a monitoring plan that can be carried out with appropriate frequency.

Although DNA metabarcoding requires validation and standardization protocols, this study shows its ability to provide an overview of the products species composition, useful to help official control authorities ensure product safety and combat unfair practices. Its application in routine assays would help verify products’ authenticity and prevent species substitution in order to satisfy one of the main objectives of the “From Farm to Fork” strategy of the European Union’s Green Deal.

## Figures and Tables

**Table 1:** Summary of label information and molecular identification. The relative read abundances were expressed in % for both animal and plant sequencing.

Sample ID	Name of the Food	Packaging	Ingredients Declared		Replicates ID	Molecular Identifications	
			Animal	Plant		Animal	Plant
S	Sausage salt and pepper	Prepacked	Swine	Flavourings Pepper Spices	S1	*Sus scrofa* (100%)	*Pisum sativum* (93%) *Piper* spp. (6.6%) *Trifolium* spp. (0.3%) Apioideae (0.04%) *Beta* spp. (0.01%)
					S2	*Sus scrofa* (100%)	*Pisum sativum* (87.2%) *Piper* spp. (12.5%) *Trifolium* spp. (0.3%) Apioideae (0.04%) *Beta* spp. (0.01%)
M	Minced adult beef	Vacuum-packed	Bovine	Natural Flavourings	M1	*Bos taurus* (69.9%) *Ovis aries* (29.6%) Murinae (0.32%) Caprinae (0.09%) *Sus scrofa* (0.13%)	Apioideae (92%) *Beta* spp. (5%) *Allium* spp. (2%) *Pisum sativum* (0.9%)
					M2	*Bos taurus* (79.8%) *Ovis aries* (19.8%) Murinae (0.33%) Caprinae (0.06%)	Apioideae (95.5%) *Beta* spp. (2.3%) *Allium* spp. (1.3%) *Pisum sativum* (0.9%)

## Data Availability

The original contributions presented in the study are included in the article and Supplementary Material, further inquiries can be directed to the corresponding author.

## Supplementary Materials

The following supporting information can be downloaded at: https://www.mdpi.com/article/10.3390/foods13182941/s1, Table S1: Number of assigned reads for each animal taxa. Table S2: Number of assigned reads for each plant taxa.
