# Supplementary material for: DNA Metabarcoding Approach as a Potential Tool for Supporting Official Food Control Programs: A Case Study
Source: Foods. 2024 Sep 17;13(18):2941. doi: 10.3390/foods13182941 (PMC11430886; doi:10.3390/foods13182941)
Supplement: Supplementary file 1 [file foods-13-02941-s001.zip › Supplementary_tables.pdf]

Supplementary Table S1. Number of assigned reads for each animal taxa.

**Molecular identification**	**Total abundance**	**S1**	**S2**	**M1**	**M2**
Sus scrofa	359019	173128	185716	175	0
Bos taurus	177494	0	0	98084	79410
Ovis aries	61244	0	0	41527	19717
Murinae	780	0	0	453	327
Caprinae	183	0	0	120	63

Supplementary Table S2. Number of assigned reads for each plant taxa.

**Molecular identification**	**Total abundance**	**S1**	**S2**	**M1**	**M2**
Pisum sativum	180226	80734	99480	7	5
Piper spp.	20008	5760	14248	0	0
Apioideae	1314	37	40	728	509
Trifolium	553	242	311	0	0
Beta spp.	75	11	12	40	12
Allium spp.	23	0	0	16	7
